# Flax and Sorghum: Multi-Element Contents and Nutritional Values within 210 Varieties and Potential Selection for Future Climates to Sustain Food Security

**DOI:** 10.3390/plants11030451

**Published:** 2022-02-06

**Authors:** Gokhan Hacisalihoglu, Paul R. Armstrong

**Affiliations:** 1 Department of Biological Sciences, Florida A&M University, Tallahassee, FL 32307, USA; 2 USDA-ARS Center for Grain and Animal Health Research, Manhattan, KS 66502, USA; paul.armstrong@usda.gov

**Keywords:** food security, nutrient dense, superfood, multi minerals, health benefits, zinc, iron, gluten free, percent daily value, elevated CO_2_

## Abstract

The Dietary Guidelines for Americans recommends giving priority to nutrient-dense foods while decreasing energy-dense foods. Although both flax (*Linum usitatissimum*) and sorghum (*Sorghum bicolor*) are rich in various essential minerals, their ionomes have yet to be investigated. Furthermore, previous studies have shown that elevated CO_2_ levels could reduce key nutrients in crops. In this study, we analyzed 102 flax and 108 sorghum varieties to investigate their ionomic variations (N, P, K, Ca, Mg, S, B, Zn, Mn, Fe, Cu, and Mo), elemental level interactions, and nutritional value. The results showed substantial genetic variations and elemental correlations in flax and sorghum. While a serving size of 28 g of flax delivers 37% daily value (DV) of Cu, 31% of Mn, 28% of Mg, and 19% of Zn, sorghum delivers 24% of Mn, 16% of Cu, 11% of Mg, and 10% of Zn of the recommended daily value (DV). We identified a set of promising flax and sorghum varieties with superior seed mineral composition that could complement breeding programs for improving the nutritional quality of flax and sorghum. Overall, we demonstrate additional minerals data and their corresponding health and food security benefits within flax and sorghum that could be considered by consumers and breeding programs to facilitate improving seed nutritional content and to help mitigate human malnutrition as well as the effects of rising CO_2_ stress.

## 1. Introduction

There are several diet-related chronic diseases (e.g., diabetics, heart disease, obesity, and cancer), and therefore, specifically plant-based nutrition is expected to be increasingly important worldwide for prevention and control of these diseases [1]. Therefore, one of the utmost research areas of plant biology has been plant nutritional values for the human diet.

**Figure 1:**
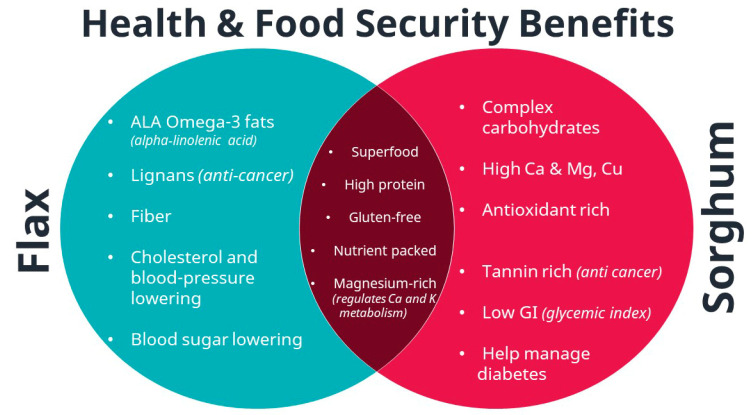
Potential health and food security benefits of flax and sorghum seeds.

Due to the growing popularity and demand for plant nutrition, there is an increasing need for research on the improvement of yield and quality of crop plants. Flax (*Linum usitatissimum*) is an annual crop and an important source of alpha linolenic acid (ALA) omega-3 fats as well as protein with all nine essential amino acids, except lysine (Figure 1) [2]. Flax is grown in cool climates including Canada, China, Russia, and the United States (North Dakota and Minnesota) [1]. Sorghum (*Sorghum bicolor*) is one of the Poaceae family cereal crops with important antidiabetic, anticholesterol, and low glycemic index (GI) features and is grown in the United States, India, Mexico, and China (Figure 1) [3]. Flax and sorghum seeds both contain a diverse set of mineral nutrients together with protein, oil, and carbohydrate (Figure 1). Ionomic profiling of the accumulated elements in living organisms has been successfully applied to study leaves, roots, whole plants, and seeds [4]. Furthermore, mineral and trace elements have been successfully determined in many other crop species including common beans, peas, soybeans, and maize [5,6,7,8,9,10,11,12,13].

Atmospheric carbon dioxide (CO_2_) levels have increased from 278 μmol/mol to 417 μmol/mol (present, 2021) and are expected to reach 550 μmol/mol and 800+ μmol/mol by 2050 and 2100, respectively, with the current average increase rate of 2.5 μmol/mol [14]. A continuous rise in the levels of atmospheric CO_2_ is expected to potentially affect plant life negatively. A study with soybean found that elevated CO_2_ levels influenced seed nutritional levels by decreasing most mineral content including the concentrations of K, Mg, Fe, and B [15]. Furthermore, a reduction in seed nitrogen (N) has been reported, and therefore, protein levels under elevated CO_2_ levels [16].

One way to mitigate future climate effects and maintain food sustainability is to screen and identify top varieties that can naturally offer superior mineral concentrations. The importance of seeds in maintaining human health and diet could be determined by their nutritional content through a recommended percent daily value (% DV, how much it contributes to a daily 2000 calorie diet) [6]. Therefore, measurement of mineral element contents can provide valuable information for consumers and crop breeders.

Among several studies that have been conducted on seed ionome, most of them have been carried out in major staple crop plants. The specific aims of this study were: (1) to determine the variability of macronutrient and trace-element concentrations among 102 flax and 108 sorghum varieties; (2) to analyze the elemental interrelationships and % DV of nutrients; (3) to identify superior varieties that could be used to improve the nutritional value potential in flax and sorghum.

## 2. Materials and Methods 

### 2.1. Flax and Sorghum Seeds

A total collection of diverse global varieties including 102 flax (*Linum usitatissimum*) and 108 sorghum (*Sorghum bicolor*) varieties were selected based on their maximum geographic diversity obtained from the USDA National Germplasm Center. All seeds used in this study were field grown with standard agronomical practices (Table 1 and Table 2).

### 2.2. Multi-Elemental Seed Analysis

Elemental concentrations of macro- and micronutrients were quantified by Waters Agricultural Labs Inc. (Camilla, GA, USA). Minerals were analyzed by open vessel wet digestion using an inductively coupled argon plasma spectrometer (ICAP, DigiBlock 3000 ICP-MS). Briefly, seeds were dried at 80 °C in an oven overnight, and then ground in a Wiley mill. A 0.5 g dried sample was mixed with 5 mL concentrated nitric acid and incubated at 95 °C for 90 min. Then, 4 mL of 30% H2O2 was added to each tube and incubated at 95 °C for 20 min. Samples were cooled for 2 min, brought to 50 mL with distilled H2O, and mixed. Samples were transferred to ICP tubes for analysis, in accordance with manufacturer’s specifications. The ICP-MS was calibrated using distilled H2O as a blank and two plant standards.

Total nitrogen (N) determination was performed by U.S. Department of Agriculture (Manhattan, KS, USA) using combustion gas analysis (LECO FP-628, St. Joseph, MI, USA), following the manufacturer’s instructions as previously described in Hacisalihoglu et al. [13].

### 2.3. Estimating Nutritional Value (% DV)

The nutritional values of flax and sorghum seeds were estimated using a 28 g dry weight serving portion. The U.S. recommended daily value indices (2000 calorie diet for adults) were as follows: Mn (2 mg), P (1000 mg), Cu (2 mg), Fe (18 mg), Mg (400 mg), Zn (15 mg), and Ca (1000 mg) [17,18]. Percent daily values (% DV) were estimated from a 28 g of seeds serving (dry weight basis) by using the following formula:

% DV = (amount of nutrient mg / recommended DV mg)∗100(1)

%DV; Percent daily values; mg; milligrams. 

### 2.4. Data Analysis

All lab analyses were completed with three replications. Elemental statistical correlation analysis was performed using SigmaPlot (SPSS Inc., Chicago, IL, USA), as described previously [9]. Descriptive statistics for each macro- and micronutrients and varieties were determined using the average of the ICP-MS results from the three biological replications. Graphs were made with SigmaPlot software (SPSS Inc., Chicago, IL, USA).

## 3. Results

### 3.1. Variations in Flax and Sorghum Multi-Element Contents

**Figure 2:**
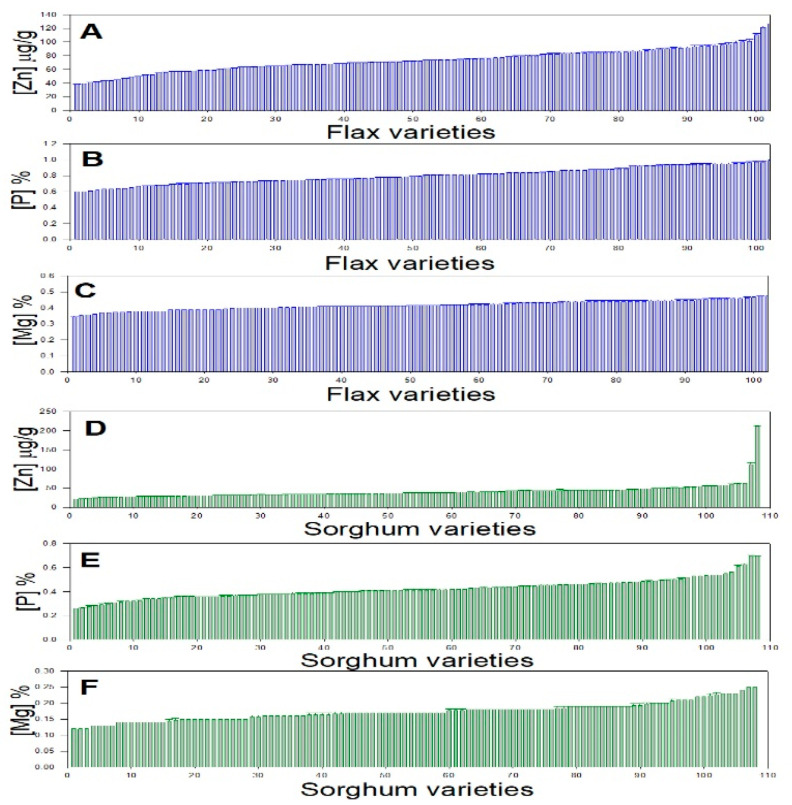
Average concentration of three replicates: (**A**) Flax Zn; (**B**) flax P; (**C**) flax Mg; (**D**) sorghum Zn; (**E**) sorghum P; and (**F**) sorghum Mg. (see Table 1 for all others).

The 102 flax varieties showed a wide variation in seed multi-element contents (Table 1 and Table 2 and Figure 2). There was a 5.7-fold range of copper (Cu) content, 4.5-fold range of iron (Fe) content, 4.2-fold range of boron (B) content, 3.3-fold range of zinc (Zn) content, 2.6-fold range of manganese (Mn) content, 2.1-fold range of calcium (Ca) content, and 2-fold range of potassium (K) and molybdenum (Mo) contents (Table 2).

The 108 flax varieties showed a wide variation in seed multi-element contents (Table 2 and Figure 2). There was a 46-fold range of Fe content, 12-fold range of Cu content, 6.6-fold range of B content, 6.3-fold range of Mn content, 9.7-fold range of Zn content, 4.5-fold range of Mo content, 5-fold range of Ca, 2.7-fold range of P, and 2.6-fold range of potassium (K) (Table 2).

**Table 1:** Mean elemental concentrations of 102 flax varieties as % (N, P, K, Mg, Ca, and S) and μg/g (Zn, Mn, Fe, Cu, and Mo) obtained from ICP-MS.

Flax Variety	N	P	K	Mg	Ca	S	B	Zn	Mn	Fe	Cu	Mo
Ames8040	3.36	0.82	0.75	0.46	0.23	0.27	17.7	93.5	23.8	87.4	10.4	3.06
Ariane	4.37	0.93	0.86	0.44	0.20	0.30	18.7	93.4	21.7	71.5	15.5	2.64
Beladiy6903	3.82	0.81	0.78	0.44	0.21	0.29	18.5	70.4	21.1	67.1	9.53	2.91
Benvenotolabrador	3.91	0.76	0.73	0.45	0.22	0.27	18.0	72.1	28.9	75.0	8.38	2.85
Charurraolajlen19	3.66	0.77	0.72	0.43	0.22	0.26	21.0	78.8	23.7	69.4	11.9	2.79
Charurraolajlen29	3.87	0.71	0.77	0.41	0.20	0.25	15.7	70.7	20.9	67.5	7.54	3.11
CIli1319	3.41	0.81	0.81	0.45	0.19	0.25	15.8	67.7	19.9	62.6	8.97	2.85
CIli1339	4.00	0.78	0.81	0.42	0.20	0.28	14.9	49.3	21.8	65.6	12.0	2.89
CIli1340	3.52	0.83	0.74	0.44	0.24	0.29	20.7	71.2	26.4	71.5	14.9	2.72
CIli1341	4.14	0.71	0.68	0.41	0.22	0.29	20.6	64.3	20.6	65.8	9.05	2.79
CIli1350	3.73	0.73	0.71	0.41	0.22	0.27	15.5	38.0	20.1	67.3	9.19	2.97
CIli1351	4.16	0.75	0.72	0.43	0.24	0.29	18.2	64.1	21.7	71.1	8.13	2.92
CIli1354	4.11	0.73	0.71	0.41	0.21	0.27	15.7	41.1	21.0	65.2	9.30	3.05
CIli1369	4.08	0.74	0.68	0.42	0.22	0.29	20.5	73.5	19.9	62.5	14.2	2.88
CIli1370	3.47	0.86	1.08	0.38	0.30	0.27	12.3	75.6	24.3	81.4	12.2	2.60
CIli1373	3.37	0.94	1.18	0.42	0.32	0.26	10.2	56.9	23.6	83.1	11.4	3.37
CIli1374	3.58	0.87	0.87	0.47	0.24	0.27	17.4	60.4	38.8	64.3	12.8	3.81
CIli1395	3.61	0.82	0.87	0.43	0.22	0.28	16.3	43.8	19.9	64.4	8.65	3.24
CIli1397	3.59	0.95	1.07	0.40	0.34	0.26	9.61	52.1	28.2	74.2	10.5	3.25
CIli1404	3.73	0.84	0.82	0.44	0.24	0.28	20.8	63.2	23.9	54.5	15.1	2.82
CIli1418	4.34	0.97	0.88	0.39	0.38	0.29	11.7	95.2	30.5	95.0	16.2	3.05
CIli1426	4.20	0.78	0.81	0.41	0.21	0.28	16.8	43.6	21.7	59.9	8.85	3.23
CIli1427	3.91	0.83	0.89	0.42	0.27	0.29	11.2	63.1	18.8	71.7	8.38	3.84
CIli1429	3.88	0.93	1.10	0.40	0.32	0.27	11.0	83.1	26.3	84.1	14.38	2.27
CIli1431	4.20	0.81	0.82	0.42	0.23	0.29	15.6	44.8	21.8	65.5	9.49	2.75
CIli1436	4.36	0.74	0.76	0.39	0.24	0.30	25.3	81.5	28.5	74.0	15.5	2.96
CIli1449	3.69	0.87	0.86	0.44	0.25	0.26	15.5	54.9	21.8	43.7	12.3	2.69
CIli1452	4.55	0.70	0.73	0.41	0.22	0.29	20.2	63.7	22.7	73.4	9.96	3.22
CIli1458	3.94	0.81	0.78	0.43	0.22	0.26	17.6	52.2	22.0	49.5	12.5	3.04
CIli1476	4.11	0.89	0.79	0.46	0.18	0.29	16.5	86.4	23.9	81.8	9.75	2.35
CIli1492	3.86	0.64	0.72	0.40	0.23	0.27	19.2	39.0	17.0	48.3	10.2	2.92
CIli1669	4.26	0.67	0.81	0.40	0.22	0.30	23.0	57.1	28.4	70.6	6.79	2.86
CIli1751	3.63	0.71	0.80	0.38	0.22	0.28	22.7	84.9	28.9	64.3	15.6	3.47
CIli1763	4.32	0.94	0.93	0.42	0.25	0.30	16.5	87.1	27.9	68.7	9.51	2.87
CIli1821	3.26	0.62	0.60	0.36	0.22	0.26	40.5	78.0	30.3	54.1	24.2	3.17
CIli1836	3.75	0.74	0.83	0.41	0.22	0.28	18.9	47.2	23.8	51.7	10.7	2.98
CIli1931	3.40	0.67	0.68	0.39	0.19	0.26	34.0	79.7	25.8	67.8	17.7	3.21
CIli1938	3.50	0.60	0.67	0.38	0.22	0.25	32.0	74.3	30.2	63.0	14.7	3.11
CIli1943	3.69	0.70	0.70	0.40	0.23	0.26	31.2	83.2	30.1	62.7	15.5	3.29
CIli1955	3.30	0.71	0.73	0.39	0.24	0.26	31.0	86.7	31.1	62.2	16.4	3.34
CIli1980	3.27	0.76	0.85	0.43	0.26	0.25	27.5	68.4	24.8	61.8	12.3	2.87
CIli1983	3.87	0.89	1.12	0.41	0.28	0.26	21.4	91.7	21.0	76.3	5.17	3.47
CIli1989	3.65	0.72	0.81	0.43	0.22	0.26	22.1	69.6	24.5	63.9	10.0	3.41
CIli1990	3.32	0.78	0.75	0.46	0.20	0.26	23.7	76.9	23.7	71.9	9.41	2.95
CIli2010	3.79	0.93	0.83	0.47	0.24	0.26	14.4	73.7	27.6	65.8	12.7	3.18
CIli2033	3.88	0.82	0.76	0.44	0.24	0.27	20.4	67.1	34.5	196.1	14.1	3.01
CIli2070	3.98	0.81	0.75	0.40	0.22	0.27	14.6	71.0	19.1	70.7	12.4	3.11
CIli2424	4.21	0.82	0.76	0.45	0.24	0.28	18.4	75.5	22.3	72.1	8.70	2.78
CIli2443	4.27	0.74	0.79	0.41	0.23	0.28	18.6	58.6	21.9	61.8	8.52	3.00
CIli2444	3.94	0.81	0.83	0.42	0.29	0.30	22.1	82.3	26.3	84.4	15.4	2.72
CIli2446	4.18	0.87	0.77	0.46	0.25	0.26	14.1	55.9	24.9	69.0	8.60	2.76
CIli2534	4.41	0.74	0.71	0.40	0.23	0.28	15.5	67.5	19.6	77.8	11.0	2.50
CIli3242	4.22	0.93	0.84	0.42	0.24	0.30	17.9	93.8	31.4	89.1	11.6	2.70
CIli3246	4.48	0.86	0.90	0.41	0.28	0.27	15.5	73.2	31.1	57.7	7.74	2.83
CIli3303	3.96	0.77	0.78	0.42	0.25	0.28	21.2	79.2	32.3	76.0	8.50	2.74
CIli3310	4.33	0.73	0.70	0.42	0.20	0.28	17.4	49.7	21.9	56.7	12.5	3.01
CIli3312	3.96	0.68	0.69	0.39	0.23	0.27	21.8	70.5	27.4	71.2	8.90	2.39
CIli3314	4.49	0.75	0.80	0.42	0.23	0.28	20.2	69.8	29.3	70.8	10.5	2.57
CIli3317	4.11	0.61	0.70	0.38	0.27	0.27	19.4	73.9	19.7	61.3	11.2	3.43
CIli3318	4.36	0.69	0.80	0.37	0.22	0.27	27.7	79.5	21.8	75.3	11.0	3.39
CIli452	4.41	0.88	0.87	0.44	0.24	0.29	13.7	100.4	34.0	92.9	7.53	2.41
CIli641	3.87	0.97	0.88	0.46	0.22	0.28	13.7	69.6	27.3	63.1	12.9	3.30
CIli642	4.11	0.79	0.77	0.41	0.25	0.27	13.0	61.2	23.0	53.3	12.2	3.09
CIli643	4.01	0.94	1.01	0.42	0.25	0.28	13.6	127.1	15.5	80.6	6.53	3.70
Coeruleum	4.02	0.92	0.95	0.40	0.30	0.28	14.1	91.2	28.1	95.3	15.4	2.54
CrownCanada	3.69	0.97	0.96	0.40	0.33	0.27	13.2	110.4	25.0	94.8	16.8	3.03
Danese129a	4.08	0.71	0.73	0.39	0.21	0.28	22.9	70.1	25.6	69.6	9.65	2.75
Dufferin	4.11	0.74	0.70	0.42	0.23	0.29	19.1	75.0	26.9	79.7	13.8	2.67
Flanders	3.72	0.72	0.67	0.41	0.23	0.27	19.0	41.8	19.4	47.4	15.1	2.91
FP966	3.61	0.63	0.72	0.39	0.25	0.25	29.3	66.2	24.5	69.7	13.6	3.52
Gercello	4.77	0.95	0.91	0.44	0.26	0.28	16.8	62.1	28.1	69.2	14.8	3.10
Giza139a	4.11	0.64	0.65	0.38	0.26	0.28	19.4	58.8	22.7	66.6	6.17	2.72
Gujrat2	2.90	0.63	0.66	0.37	0.24	0.24	29.7	77.3	37.3	57.9	15.8	3.16
H39seln	3.97	0.70	0.68	0.39	0.25	0.28	18.9	58.7	24.8	61.9	6.13	2.44
Hazeldean	4.44	0.95	0.81	0.44	0.21	0.28	14.8	94.7	23.2	82.7	13.1	2.93
Jalaun	3.90	0.76	1.00	0.37	0.29	0.26	15.9	67.6	21.5	59.6	5.72	2.39
Jalomita	4.27	0.87	0.75	0.44	0.23	0.28	16.3	84.5	20.9	73.9	14.2	2.86
Katan92	4.22	0.77	0.67	0.43	0.21	0.27	17.8	68.0	20.1	57.4	11.0	2.96
Katan93	3.63	0.81	0.71	0.41	0.21	0.27	17.5	83.6	24.7	67.1	12.4	3.08
Kenyaci709	3.90	0.73	0.62	0.34	0.22	0.27	21.1	90.3	41.0	66.5	14.0	2.92
Mcduffp900	3.58	0.65	0.66	0.38	0.28	0.27	17.4	67.5	23.2	57.7	12.0	3.39
Moose	3.97	0.93	0.87	0.46	0.24	0.28	15.1	71.6	26.3	60.5	11.9	3.26
NP80	3.74	0.60	0.59	0.35	0.20	0.25	25.0	85.6	30.8	53.5	16.9	2.97
NO1040	3.73	0.81	0.74	0.47	0.23	0.28	20.1	75.0	32.4	65.7	14.3	3.05
NO11	3.58	0.95	0.88	0.39	0.37	0.27	11.0	89.3	33.8	70.0	16.2	2.86
NO129	4.98	0.78	0.74	0.41	0.24	0.30	19.7	57.1	21.4	65.9	7.79	3.12
Norland	4.05	0.95	0.87	0.44	0.24	0.27	16.2	101.3	20.4	81.5	13.2	2.60
NP121	4.32	0.91	1.11	0.40	0.25	0.27	23.9	121.1	18.9	74.8	4.21	3.58
NP124	3.99	0.79	0.82	0.44	0.23	0.26	20.0	64.7	23.0	56.5	10.2	2.53
Omega	3.86	0.99	0.95	0.43	0.37	0.27	13.0	99.1	28.6	80.3	19.4	4.58
Pasrur2	2.91	0.67	0.63	0.37	0.24	0.24	26.0	84.5	34.5	65.9	16.0	3.05
Rembrandt	3.63	0.83	0.74	0.44	0.22	0.28	19.1	90.9	23.1	78.8	12.7	2.38
Saidabad	4.19	0.75	0.70	0.41	0.22	0.29	19.8	96.8	32.3	88.4	18.9	2.99
Somme	3.84	0.86	0.81	0.44	0.23	0.26	17.8	84.5	23.6	68.9	17.4	3.08
SzeepiOlajlen	4.11	0.92	0.87	0.36	0.37	0.27	11.4	73.3	25.2	70.3	13.9	2.85
Tomagaon	3.53	0.76	0.80	0.42	0.24	0.25	11.0	66.8	20.7	59.3	7.90	2.39
Uruguay	3.80	0.84	0.72	0.43	0.21	0.27	17.3	84.2	25.5	64.2	13.6	2.80
Verin	3.69	0.84	0.82	0.44	0.23	0.27	18.0	87.9	24.2	81.0	12.1	2.83
Viking	4.17	0.85	0.69	0.44	0.27	0.27	15.8	57.7	36.6	49.7	8.78	3.12
Vimy	4.00	0.68	0.97	0.41	0.27	0.28	23.0	64.9	35.8	76.3	9.59	2.93
W62611FKA14	3.74	0.92	0.79	0.45	0.20	0.27	15.8	83.6	22.0	74.3	11.4	2.98
WickingHeggenen	4.15	0.87	0.71	0.45	0.25	0.27	19.0	82.5	34.4	66.3	11.5	2.96

**Table 2:** Mean elemental concentrations of 108 sorghum varieties as % (N, P, K, Mg, Ca, and S) and μg/g (Zn, Mn, Fe, Cu, and Mo) obtained from ICP-MS.

Sorghum Variety	N	P	K	Mg	Ca	S	B	Zn	Mn	Fe	Cu	Mo
52	2.99	0.70	0.58	0.25	0.05	0.43	10.3	43.3	50.9	91.5	11.3	2.65
282	2.19	0.48	0.46	0.19	0.02	0.17	5.07	47.3	25.5	267	8.41	2.32
434	2.28	0.70	0.54	0.25	0.05	0.39	9.97	60.9	50.2	235	16.6	3.52
1398	2.78	0.61	0.74	0.23	0.05	0.38	13.0	59.8	61.1	643	13.4	2.62
1491	1.94	0.47	0.47	0.20	0.01	0.15	2.80	45.7	26.1	269	3.37	2.02
1728	1.80	0.46	0.60	0.16	0.02	0.14	7.27	211	34.6	49.4		2.09
3967	1.51	0.41	0.47	0.18	0.01	0.14	3.05	43.3	18.1	35.6	3.83	2.10
4058	1.66	0.40	0.44	0.16	0.02	0.15	2.53	44.5	19.2	33.6	3.40	1.15
4080	2.02	0.49	0.53	0.19	0.02	0.15	3.16	36.7	20.8	32.6	3.15	1.46
4116	1.80	0.36	0.37	0.14	0.02	0.15	2.94	37.5	16.6	30.6	3.23	1.79
93447	2.02	0.41	0.53	0.17	0.04	0.12	3.54	55.7	21.9	44.2	27.3	2.06
54K94	2.21	0.63	0.72	0.21	0.02	0.16	4.69	43.4	35.1	55.3	6.37	1.81
88-07095	1.51	0.34	0.47	0.12	0.01	0.11	9.37	21.8	17.4	21.8	3.41	1.47
88-07105	1.60	0.26	0.37	0.12	0.02	0.14	2.40	29.7	15.6	35.8	4.92	1.34
88-07108	1.78	0.32	0.40	0.16	0.02	0.14	2.91	112	22.6	45.3	6.37	1.22
88-07197	2.01	0.41	0.41	0.19	0.02	0.16	2.89	31.4	17.5	44.7	6.55	1.76
88-07207	2.06	0.53	0.54	0.18	0.02	0.14	2.73	37.2	26.7	34.0	5.13	1.66
88-07225	1.99	0.54	0.63	0.17	0.02	0.16	2.64	48.1	27.8	45.2	6.82	1.99
A7774	2.78	0.56	0.57	0.21	0.02	0.18	3.31	63.0	18.7	53.5	4.58	2.43
A84	1.45	0.42	0.53	0.18	0.02	0.13	4.12	37.6	15.9	37.5	3.18	2.92
A96	2.12	0.46	0.48	0.19	0.03	0.14	3.64	35.4	18.8	42.1	4.11	2.60
ABTx631	1.48	0.38	0.41	0.17	0.02	0.12	3.16	30.0	15.2	29.6	4.22	2.96
AcchoKaruha	2.22	0.47	0.42	0.18	0.02	0.12	3.04	44.8	25.2	55.3	4.61	3.19
AS4055	1.49	0.37	0.49	0.15	0.01	0.12	3.11	27.3	11.7	24.9	3.31	2.17
AS4136	1.96	0.42	0.49	0.16	0.01	0.15	2.61	44.6	20.0	34.6	4.42	3.72
AS5826	2.06	0.40	0.43	0.18	0.02	0.14	3.22	42.7	15.9	20.6	5.89	3.35
Barking119	2.01	0.44	0.53	0.18	0.01	0.13	3.23	44.6	19.0	36.6	5.22	2.38
BE25	2.19	0.48	0.48	0.18	0.02	0.14	3.55	44.2	21.8	45.0	7.27	3.09
Bok11	1.68	0.43	0.44	0.19	0.02	0.15	3.60	36.0	17.2	38.0	4.00	3.91
BrownKaoliang	2.17	0.42	0.47	0.17	0.02	0.15	5.06	43.6	16.3	51.7	5.47	2.40
BTx623	1.81	0.37	0.49	0.15	0.02	0.10	3.78	23.3	13.0	39.0	5.15	1.48
ChananSingoo	1.99	0.43	0.43	0.18	0.03	0.14	3.76	51.0	20.5	33.8	6.16	3.14
ChineseAmber	2.63	0.53	0.45	0.23	0.02	0.17	5.76	57.1	28.6	46.1	7.17	2.94
Collier	1.73	0.41	0.39	0.17	0.01	0.17	4.04	24.2	21.1	32.3	3.97	2.23
Cowley	1.83	0.31	0.31	0.15	0.03	0.15	5.42	31.3	14.5	29.6	4.61	4.10
DaShanDong	1.69	0.47	0.53	0.18	0.02	0.13	3.80	39.9	17.9	35.0	4.24	2.52
Dokhnah	2.17	0.40	0.36	0.17	0.01	0.15	6.74	44.6	22.2	38.0	4.66	1.82
Elmota	1.92	0.46	0.43	0.20	0.02	0.14	5.13	43.1	18.7	38.8	6.29	2.46
ERJieZi	1.58	0.34	0.43	0.13	0.01	0.12	3.13	33.5	18.5	29.3	3.93	1.72
FAO54919	2.11	0.46	0.40	0.19	0.01	0.16	2.96	43.4	18.8	33.8	4.18	2.00
Grif534	1.63	0.26	0.34	0.13	0.02	0.13	2.08	35.7	16.6	40.0	5.67	1.48
Grif539	1.87	0.30	0.32	0.14	0.03	0.14	2.52	37.4	21.5	42.0	5.75	1.55
Grif553	1.89	0.32	0.33	0.14	0.01	0.15	2.08	28.4	19.6	35.7	5.83	1.48
Grif574	1.95	0.48	0.41	0.20	0.03	0.16	3.95	55.2	26.0	48.4	6.99	1.53
Grif604	1.74	0.29	0.28	0.14	0.02	0.14	2.28	31.2	9.7	49.9	5.02	1.61
Grif610	1.63	0.28	0.34	0.14	0.03	0.14	2.59	48.9	15.2	57.0	7.19	1.49
Grif7260	1.57	0.42	0.58	0.15	0.02	0.13	4.94	30.2	15.4	33.0	3.80	2.01
Grif7263	1.82	0.39	0.44	0.17	0.01	0.13	2.66	33.0	13.7	38.6	5.07	2.27
IS1019	2.25	0.31	0.41	0.14	0.02	0.14	5.73	34.0	17.7	28.7	4.58	1.85
IS10931	2.31	0.46	0.44	0.18	0.02	0.17	3.02	33.2	19.1	43.2	4.32	2.91
IS1213C	1.98	0.36	0.36	0.17	0.02	0.13	4.66	29.1	20.0	35.7	4.13	2.59
IS12684C	1.88	0.37	0.54	0.16	0.03	0.14	4.98	37.5	18.7	40.6	6.01	2.35
IS12845	2.06	0.44	0.44	0.19	0.04	0.13	5.76	39.2	29.2	38.6	6.52	1.43
IS13232	1.76	0.49	0.56	0.17	0.02	0.14	3.68	30.9	24.9	42.5	4.07	2.06
IS13236	2.20	0.50	0.62	0.17	0.03	0.15	3.63	32.6	32.0	41.9	6.32	1.92
IS14098	1.67	0.36	0.43	0.15	0.03	0.13	3.48	35.0	13.3	32.8	3.80	2.02
IS24424	2.24	0.52	0.49	0.23	0.03	0.14	4.94	47.5	25.4	47.7	4.39	1.78
IS24449	1.77	0.40	0.42	0.17	0.02	0.12	3.40	33.0	13.0	32.8	3.15	2.30
IS24451	1.97	0.41	0.34	0.19	0.01	0.14	2.68	37.4	16.3	32.8	4.20	2.27
IS27569	1.69	0.42	0.47	0.16	0.01	0.12	2.95	36.7	15.5	48.8	4.14	2.36
IS27601	1.43	0.37	0.52	0.15	0.02	0.14	3.67	38.7	19.2	14.0	4.24	1.88
IS28214	1.76	0.43	0.54	0.16	0.02	0.13	2.92	49.6	18.8	35.4	4.02	1.98
IS2871C	2.23	0.53	0.50	0.22	0.02	0.14	5.50	52.7	17.1	42.0	5.36	2.30
IS2874	1.87	0.45	0.46	0.19	0.03	0.14	3.04	48.0	13.9	45.0	4.16	2.26
IS3098	1.43	0.38	0.47	0.15	0.02	0.12	3.37	29.6	11.5	28.5	2.32	1.83
IS5168C	2.16	0.45	0.41	0.21	0.03	0.14	4.19	51.4	22.9	38.3	5.54	2.67
IS6541	2.13	0.41	0.32	0.18	0.02	0.14	3.25	35.6	14.6	37.1	3.67	1.59
IS6733C	1.79	0.37	0.47	0.15	0.01	0.13	3.50	40.4	16.2	34.5	4.03	2.58
IS8120C	1.91	0.34	0.38	0.14	0.01	0.12	2.72	22.2	12.7	19.3	3.43	2.42
JolaNandyal	1.72	0.36	0.41	0.13	0.02	0.12	3.63	29.1	13.4	25.2	2.71	1.78
JowarRedJan	2.06	0.38	0.39	0.16	0.02	0.12	6.00	38.1	22.3	27.2	3.35	2.24
KA12Janjari	1.84	0.41	0.69	0.18	0.02	0.14	5.03	32.6	15.5	28.0	5.38	2.73
Kabutuwa	1.96	0.51	0.56	0.21	0.03	0.14	5.24	57.6	25.2	24.4	7.93	5.12
Kaoliang	2.05	0.41	0.46	0.16	0.02	0.13	3.70	34.2	12.8	27.4	3.90	3.10
Kaoliangwx	1.48	0.38	0.43	0.15	0.02	0.13	3.16	35.1	13.9	34.7	4.11	3.26
KharuthWara	2.18	0.36	0.33	0.18	0.03	0.13	4.31	41.3	15.9	42.9	3.10	2.99
Kulum	1.61	0.38	0.45	0.14	0.02	0.12	3.27	27.1	12.9	35.1	3.61	1.75
Kuyuma	1.94	0.35	0.43	0.13	0.01	0.11	3.41	28.2	12.4	23.2	2.92	2.35
Leoti	1.70	0.38	0.44	0.18	0.01	0.13	4.52	32.7	25.2	22.1	3.33	2.06
LianTouSan	1.97	0.48	0.55	0.18	0.01	0.14	2.47	42.8	17.6	35.0	5.31	2.15
Lula	1.61	0.38	0.39	0.17	0.02	0.13	3.60	38.8	18.5	30.2	4.69	3.03
M35-1	1.68	0.32	0.36	0.16	0.01	0.12	3.42	26.7	10.5	24.9	2.19	2.24
ManfrediMinu	1.99	0.43	0.45	0.18	0.02	0.15	4.62	30.0	42.3	36.7	5.43	1.38
Marupantse	1.68	0.41	0.46	0.16	0.02	0.14	3.09	33.6	20.5	35.4	4.98	2.58
Mashica	1.97	0.43	0.52	0.17	0.02	0.13	3.75	34.1	14.9	26.5	2.51	2.79
MN1592	1.67	0.36	0.41	0.17	0.02	0.14	3.46	33.8	18.5	33.5	3.54	3.91
MN4315	2.41	0.54	0.44	0.23	0.02	0.16	7.76	44.8	22.5	42.3	6.86	2.08
MN586	1.73	0.41	0.46	0.17	0.01	0.17	5.47	38.2	16.6	34.5	6.12	3.03
MN707	1.93	0.41	0.48	0.15	0.02	0.14	3.28	26.5	20.3	21.1	4.27	3.82
MsumbjiSB117	2.07	0.47	0.41	0.18	0.02	0.14	2.62	43.5	32.7	39.0	4.56	1.62
N290b	1.71	0.36	0.47	0.16	0.02	0.12	2.63	28.5	14.0	29.7	4.06	2.86
OrangeNo1	2.16	0.28	0.31	0.12	0.02	0.14	2.53	28.4	20.7	22.6	4.27	3.92
P9517	1.83	0.39	0.42	0.17	0.02	0.14	2.03	35.9	19.5	37.1	6.55	2.33
R3	1.95	0.45	0.43	0.18	0.02	0.15	2.83	40.8	19.7	32.2	5.42	3.51
S1049	2.66	0.46	0.43	0.19	0.03	0.16	1.96	49.9	19.3	36.2	6.07	2.20
S24	2.85	0.50	0.44	0.22	0.02	0.15	4.66	43.2	21.3	37.7	9.02	1.93
SAP155	1.82	0.35	0.36	0.15	0.01	0.11	2.51	35.8	15.9	33.3	4.06	1.60
SAP157	1.55	0.36	0.38	0.17	0.02	0.12	3.74	26.3	16.1	24.6	4.32	2.37
SAP158	1.72	0.36	0.54	0.15	0.03	0.12	2.69	27.5	17.3	27.8	3.34	2.30
SAP172	1.80	0.43	0.42	0.18	0.02	0.13	2.25	37.2	14.7	36.7	4.46	2.82
SDSL87046T	1.52	0.38	0.41	0.16	0.02	0.11	2.71	31.0	15.1	29.2	4.59	1.75
Shangani9356	1.65	0.39	0.47	0.14	0.02	0.13	2.37	30.5	16.8	31.4	2.91	1.84
SO85	1.96	0.49	0.47	0.19	0.02	0.15	2.75	52.5	20.1	18.5	7.41	3.78
StFederita	2.22	0.45	0.43	0.17	0.03	0.13	3.64	29.3	22.3	27.2	6.49	1.72
Takanda	2.37	0.55	0.47	0.24	0.02	0.14	3.33	39.1	24.3	50.1	5.50	1.90
Texas660	1.93	0.44	0.35	0.20	0.02	0.14	2.05	44.0	15.9	42.1	5.54	2.52
UI4822	1.99	0.44	0.49	0.19	0.01	0.16	2.47	56.1	15.0	47.4	4.62	2.37
Wray	1.78	0.41	0.35	0.22	0.01	0.13	5.62	36.1	15.0	29.8	4.03	3.17

### 3.2. Elemental Correlations among Nutrients in Seeds

**Figure 3:**
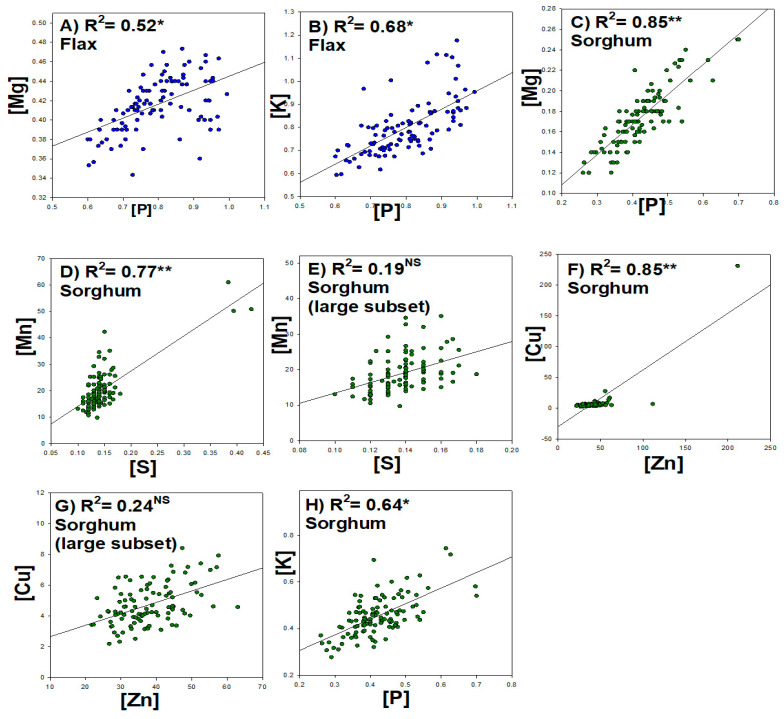
Selected correlations among seed elemental concentrations: (**A**) Flax P and Mg; (**B**) flax P and K; (**C**) sorghum P and Mg; (**D**) sorghum S and Mn; (**E**) sorghum large subset S and Mn; (**F**) sorghum Zn and Cu; (**G**) sorghum large subset Zn and Cu; (**H**) sorghum P and K. * and **, significant at the *p* < 0.05 and *p* < 0.01, respectively. NS, not significant, as determined using linear regression; R^2^, linear regression coefficient squared.

There was a positive relationship between flax seed P and Mg (Figure 3A) and a good positive correlation between P and K (Figure 3B). There was a strong positive relationship between sorghum seed P and Mg (Figure 3C) and S and Mn (Figure 3D,E). A weak relationship was observed between sorghum seed Zn and Cu (Figure 3F,G) when excluding the very high singular point. Sorghum seed P and K had a reasonable positive correlation (Figure 3H).

### 3.3. Nutritional Value of Flax and Sorghum

**Figure 4:**
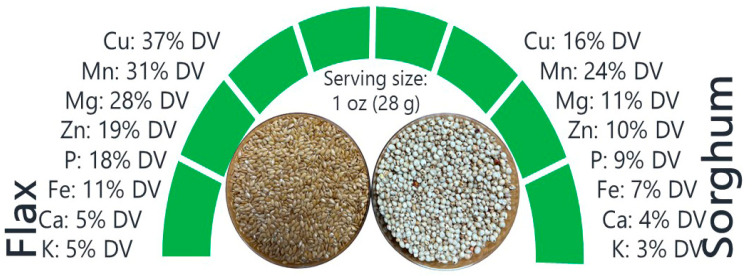
Daily value (% DV) provided by the 28 g serving size of flax and sorghum.

Eight minerals were analyzed based on the United States Department of Agriculture (USDA) [16,17] recommended percentage daily value (% DV), which is a calculation of nutritional content in a 28 g (1 oz) serving of food and contribution to a 2000 calorie daily diet (USDA, 2020) [15]. As shown in Figure 4, daily consumption of 28 g of flax seeds could provide 37% DV of Cu, 31% DV of Mn, 28% DV of Mg, 19% DV of Zn, 19% DV of Zn, 18% DV of P, 11% DV of Fe, and 5% DV of Ca and K.

Our analysis of sorghum showed that daily consumption of 28 g seeds could provide 24% DV of Mn, 16% DV of Cu, 11% DV of Mg, 10% DV of Zn, 9% DV of P, 7% DV of Fe, 4% DV of Ca, and 3% DV of K (Figure 4).

### 3.4. Identification of Promising Top Flax and Sorghum Varieties

Based on the results of screening the 102 flax varieties, six varieties were chosen for their superior mineral content performance: Omega, Clli1374, Clli1418, Clli1821, Clli643, and Clli2033 (Table 3). Similarly for screening of 108 sorghum varieties, six varieties were chosen for their superior mineral content performance: PI529799, PI365024, PI185574, PI266958, PI534144, and PI550850 (Table 3).

## 4. Discussion

The expected rising carbon dioxide (CO_2_) levels (from 416 ppm in 2021 to 550 ppm in 2050) could cause stress to food crop plants. Furthermore, this could cause a reduction in nutritional quality or fewer nutritious crops, and therefore, trigger malnutrition [15,16]. One approach to minimize this issue is to identify food crop varieties with higher natural nutrient composition potential.

In this study, we analyzed ionomic data from 210 flax and sorghum varieties collected from around the world. Our results demonstrated substantial genetic variation in both crop species (Figure 2 and Table 4). This is consistent and follows a number of recent findings in peas, soybean and common beans, pearl millet, and sweet potato [7,9,13,19,20].

A better understanding of the relationships among various mineral nutrients is also critically important. A small number of correlated element pairs were detected in the current study. The selected correlations in Figure 3 were the best examples of elemental pair correlations in both flax and sorghum. The results of our analysis of macronutrients (N, P, K, Ca, Mg, and S) and micronutrients (Zn, Fe, Cu, B, Mn, and Mo) are summarized in Figure 3. In particular, the correlation analysis showed that seed P had a positive correlation with K and Mg in both crop species, therefore, their accumulation may be related. Furthermore, sorghum seed Zn had a positive correlation with Cu, which suggests that accumulation of these trace elements is related. Furthermore, the positive correlations between element pairs suggest potential shared transport systems in flax and sorghum systems. Similar positive associations between Zn and Cu have been reported in previous studies in soybean and common beans [7,9]. These findings are also consistent with previous studies in sweet potatoes that showed medium to high correlations among minerals such as Fe, Zn, Ca, and Mg [20]. This may suggest that elemental correlations may simplify selection for future breeding efforts. Furthermore, this is consistent with studies in pearl millet that have reported good elemental correlation and the possibility of simultaneous improvement of those nutrients [19].

Overall, among the 210 total varieties, six unique flax varieties and six unique sorghum varieties were identified with superior seed nutrient composition (Table 3). In flax, the highest P and Mo were observed in variety Omega, while the highest Cu and B were observed in variety Clli1821. In sorghum, the highest K, Mn, Fe, and B were observed in variety PI365024, while the highest Mg, Ca, S, and Ni were observed in variety PI185574. In addition, four more flax and sorghum varieties were identified as superior varieties (Table 3). These sets of a few selected superior varieties show significantly higher mineral concentration, which suggests that there is a potential of further improving mineral nutrient content in both flax and sorghum. Similarly, Gorindaraj et al. [19] explored the genetic variability of pearl millet for seed nutritional traits and reported the top 10 pearl millet accessions that could be used to develop nutritionally superior cultivars.

## 5. Conclusions

In this study, the multi-element contents and nutritional values of 102 flax and 108 sorghum varieties were evaluated. Our results revealed that there is substantial genetic variation of seed mineral nutrient traits both in flax and in sorghum. We elaborated on the six superior flax varieties and the corresponding six superior sorghum varieties that seem to hold promise for mitigating rising CO2 stress as well as malnutrition. This study also provides an opportunity for future genetic studies to further efforts in biofortification efforts of flax and sorghum.

## Figures and Tables

**Table 3:** A set of top six superior varieties of flax and sorghum with superior seed mineral concentration. Highest, the highest mean for that specific element; High, considerably higher mean than other varieties.

**Flax Variety**	**Performance**
Omega	Highest: P; Mo. High: Ca, Zn, Cu, Ni
Clli1374	Highest: Mg. High: Mn, Mo
Clli1418	Highest: Ca. High: Ni, Fe, P
Clli1821	Highest: B, Cu
Clli643	Highest: Zn. High: Mo, K
Clli2033	Highest: Fe, High: Ni, Mn
**Sorghum Variety**	**Performance**
PI520799	Highest: P. High: Mg, Ca, S, B, Zn, Mn, Fe, Zn, Cu, Mo
PI365024	Highest: K, B, Mn, Fe. High: Mg, Ca, S, Zn, Cu
PI185574	Highest: Mg, Ca, S, Ni. High: P, K, B, Mn, Fe, Cu
PI266958	Highest: Zn. High: K, B, Mn, Ni
PI534144	Highest: Mo. High: Zn, Cu, P
PI550850	Highest: Cu, High: Ca

**Table 4:** Descriptive statistics in seed ionomic concentrations of 102 diverse flax genotypes and 108 sorghum genotypes. SD, standard deviation. Each value is the mean of three replicates.

**Macronutrients**							
		**N**	**P**	**K**	**Ca**	**Mg**	**S**
		%	%	%	%	%	%
**Flax**	**Avg.**	3.92	0.80	0.80	0.24	0.42	0.27
	**SD**	0.37	0.10	0.12	0.04	0.03	0.01
	**Min**	2.90	0.60	0.59	0.18	0.34	0.24
	**Max**	4.98	0.99	1.18	0.38	0.47	0.30
**Sorghum**	**Avg.**	1.93	0.42	0.45	0.02	0.17	0.15
	**SD**	0.32	0.08	0.09	0.01	0.03	0.05
	**Min**	1.35	0.26	0.28	0.01	0.12	0.10
	**Max**	2.99	0.70	0.74	0.05	0.25	0.43
**Micronutrients (Trace elements)**							
		**Zn**	**Fe**	**Cu**	**B**	**Mn**	**Mo**
		µg/g	µg/g	µg/g	µg/g	µg/g	µg/g
**Flax**	**Avg.**	73.40	70.10	11.80	18.80	25.50	2.98
	**SD**	16.90	16.50	3.45	5.36	5.11	0.35
	**Min**	38.00	43.70	4.21	9.61	15.50	2.27
	**Max**	127.00	196.00	24.20	40.50	41.00	4.58
**Sorghum**	**Avg.**	40.80	48.10	5.25	3.89	20.10	2.35
	**SD**	20.1	69.10	2.96	1.79	7.98	0.73
	**Min**	21.80	14.00	2.19	1.96	9.70	1.15
	**Max**	211	643	27.0	13.0	61.1	5.12

## Data Availability

Not applicable.
